# The Duplicated Genes Database: Identification and Functional Annotation of Co-Localised Duplicated Genes across Genomes

**DOI:** 10.1371/journal.pone.0050653

**Published:** 2012-11-28

**Authors:** Marion Ouedraogo, Charles Bettembourg, Anthony Bretaudeau, Olivier Sallou, Christian Diot, Olivier Demeure, Frédéric Lecerf

**Affiliations:** 1 INRA, UMR1348 PEGASE, Saint-Gilles, France; 2 Agrocampus OUEST, UMR1348 PEGASE, Rennes, France; 3 GenOuest Platform, INRIA/Irisa – Campus de Beaulieu, Rennes, France; Cairo University, Egypt

## Abstract

**Background:**

There has been a surge in studies linking genome structure and gene expression, with special focus on duplicated genes. Although initially duplicated from the same sequence, duplicated genes can diverge strongly over evolution and take on different functions or regulated expression. However, information on the function and expression of duplicated genes remains sparse. Identifying groups of duplicated genes in different genomes and characterizing their expression and function would therefore be of great interest to the research community. The ‘Duplicated Genes Database’ (DGD) was developed for this purpose.

**Methodology:**

Nine species were included in the DGD. For each species, BLAST analyses were conducted on peptide sequences corresponding to the genes mapped on a same chromosome. Groups of duplicated genes were defined based on these pairwise BLAST comparisons and the genomic location of the genes. For each group, Pearson correlations between gene expression data and semantic similarities between functional GO annotations were also computed when the relevant information was available.

**Conclusions:**

The Duplicated Gene Database provides a list of co-localised and duplicated genes for several species with the available gene co-expression level and semantic similarity value of functional annotation. Adding these data to the groups of duplicated genes provides biological information that can prove useful to gene expression analyses. The Duplicated Gene Database can be freely accessed through the DGD website at http://dgd.genouest.org.

## Introduction

A growing body of literature has shown that eukaryotic genomes contain groups of co-localised genes whose chromosomal location plays a role in the regulation of gene expression [1], [2], [3], [4], [5], [6], [7], [8]. Part of these groups stems from gene duplications. Although duplicated genes are initially identical, they can evolve in different ways after the duplication event [9]. Some can remain co-regulated by retaining the same *cis-*regulatory motifs whereas others acquire different patterns of expression, resulting in un-correlated gene expression or even different tissue expression patterns. There may even be discrepancies in the co-expression patterns of duplicated genes depending on the genes or species analysed. In yeast [10] and *C. elegans* [11] for example, expression patterns are more similar between two duplicated genes than between two randomly-selected genes. Conversely, there are also reports of divergent profiles between duplicated genes according to expression level [12], [13] and spatial expression [14], [15], [16], [17], [18]. Identifying groups of duplicated co-localised genes at a genomic scale for several species and characterizing both the expression and function of these genes would help bring a larger overview on this issue. While it is possible to get information on duplicated genes through a single gene query (i.e. Ensembl via its paralog genes list [19]), there is still no list of such duplicated genes available at genome-wide scale. Other tools dedicated to phylogeny studies only list duplicated genes without considering their co-location [20],[21]. In addition, none of these tools give any information on gene expression level. Therefore, many researchers are forced to identify duplicated genes in their species of interest ‘by hand’ and then aggregate functional information from different sources [22], .

This situation is further complexified by the fact that gene duplications can be divided into three major classes: 1) genomic-level duplications generated from whole genome or chromosomal duplication; 2) tandem duplications with genes closely localised in the same chromosome region; 3) other duplications corresponding to genes with distant genomic locations [31]. In addition, recent studies also show that chromatin structures play a role in the co-expression of genes (for review, see [32]), including chromatin loops [33] or chromosome pairing in RNA factories [34], [35]. Therefore, the co-location of genes may play a role in the regulation of their expression. For these reasons, we focused on tandem duplicated genes or groups of genes from multigene families (the above class 2 duplicated genes) further referred to as “groups of duplicated genes”.

Here, we identified duplicated and co-localised genes from 9 different species. Co-expression and functional similarities between these duplicated genes were also determined. All this data is available through the Duplicated Genes Database (DGD) developed by our team.

## Results

### Database Implementation

**Figure 1:**
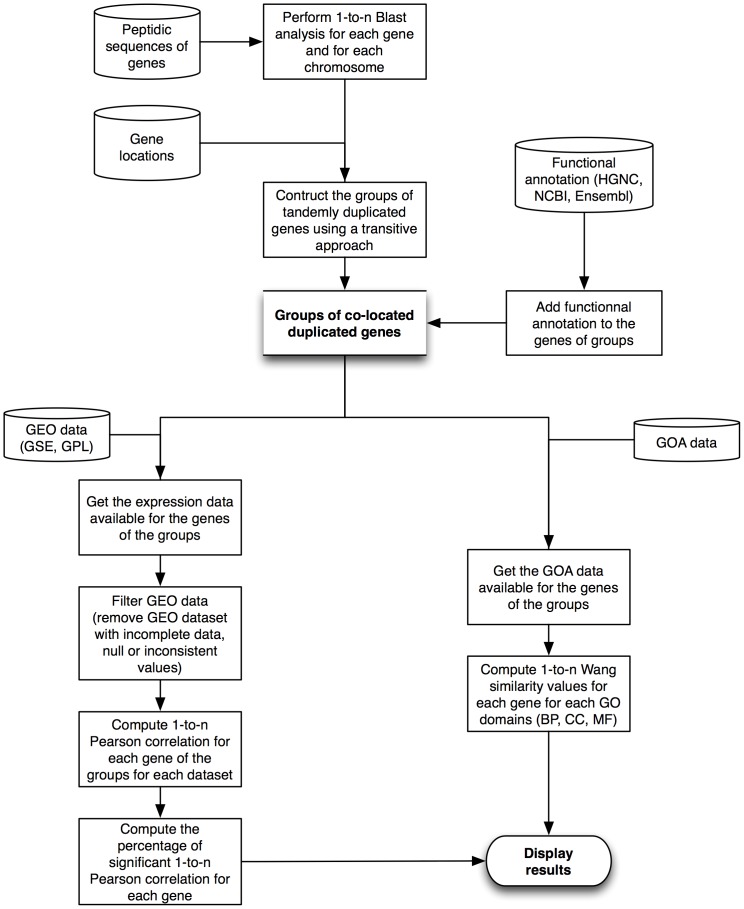
DGD workflow. Description of the DGD database development process, from sequence similarity analyses and integration of gene annotation data from NCBI, Ensembl and HGNC websites to the integration and computation of functional data from GEO (Gene Expression Omnibus) and GOA (Gene Ontology Annotation).

The DGD workflow is depicted in Figure 1. In step one of the process, pairwise BLAST analyses were performed for each gene and each chromosome. These BLAST results were used with the genomic location of the genes to determine groups of co-localised duplicated genes. Gene annotations, i.e. name and description, were also added.

In step two of the process, gene co-expression and semantic similarity of GO annotations were determined. First, GEO expression data and GO annotations were retrieved for each duplicated gene. Then, after filtering the gene expression data, pairwise Pearson correlations were computed for each pair of genes in a group for each GEO dataset. The semantic similarity value for each pair was computed using the method of Wang [36].

The DGD website outputs this data in a dynamic image linking each gene in a group to the different values available.

### Database Content

**Figure 2:**
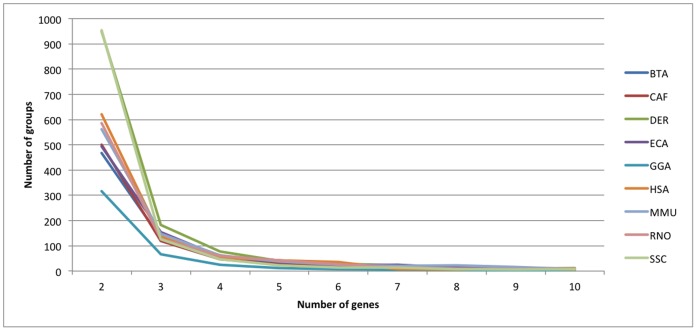
Distribution of the number of groups of duplicated genes according to number of duplicated genes. BTA: *Bos taurus*; CAF: *Canis familiaris*; DER: *Danio rerio*; ECA: *Equus caballus*; GGA: *Gallus gallus*; HSA: *Homo sapiens;* MMU: *Mus musculus*; RNO: *Rattus norvegicus* and SSC: *Sus scrofa*.

In total, the DGD contains 8411 groups of duplicated genes. By species, the number of groups varies from 444 in *Gallus gallus* (GGA) to 1412 in *Danio rerio* (DER) (Table 1). The number of duplicated genes also varies according to species, ranging from 1251 genes in GGA to 6036 in *Mus musculus* (MMU). Surprisingly, the majority of between-species variation comes from groups of 2 and 3 genes, whereas the numbers of groups of 4 and more genes are fairly similar (Figure 2). Mammalian species have similar patterns, except in *Sus scrofa* (SSC). The highest number of groups of 2 and 3 duplicated genes are found in DER (1132 groups) and SSC (1080 groups), while GGA has fewer duplicated groups than other species.

**Table 1:** Statistics on DGD content.

	HSA	MMU	RNO	CAF	GGA	BTA	DER	ECA	SSC
Total peptides	74640	40732	32948	25559	22194	26977	28630	22641	19083
Non-redundant peptides	47313	30659	24812	22383	19371	23833	26204	21551	18273
Groups	964	1008	959	751	444	798	1412	894	1229
Genes in groups	3710	6036	4899	2647	1251	3714	5830	4601	4210

For each species (*Bos taurus* (BTA), *Danio rerio* (DER), *Canis familiaris* (CAF), *Gallus gallus* (GGA), *Equus caballus* (ECA), *Homo sapiens* (HSA), *Mus musculus* (MMU), *Rattus norvegicus* (RNO) and *Sus scrofa* (SSC)), the numbers of peptide sequences used in the analyses (only non-redundant) are reorted here with the number of peptide sequences initially available (total).

There are also differences between species according to size of the groups. The median size of duplicated groups is 105 kb in humans (HSA), with other species having fairly similar values, ranging from 58 kb in GGA to 248 kb in horse (ECA) (Table 2). Mean size is 641 kb in humans, and ranges from 601 kb in pig (SSC) to 1360 kb in rat (RNO). Gene number of the largest group is 77 in humans (corresponding to a group of olfactory receptor genes), and ranges from 428 genes in *Danio rerio* (corresponding to a Zinc finger genes group) down to 62 genes in *Gallus gallus* (an unidentified genes group as no annotations were available, although the Pfam database [37] reported a keratin domain).

**Table 2:** Statistics for the groups of duplicated genes.

	HSA	MMU	RNO	CAF	GGA	BTA	DER	ECA	SSC
Mean group size (kb)	641	1007	1360	1317	892	1167	666	3368	601
Median group size (kb)	105	144	235	165	58	154	111	248	151
Maximum number of genes in largest groups	77	267	217	133	62	174	428	171	164

For each species (*Bos taurus* (BTA), *Danio rerio* (DER), *Canis familiaris* (CAF), *Gallus gallus* (GGA), *Equus caballus* (ECA), *Homo sapiens* (HSA), *Mus musculus* (MMU), *Rattus norvegicus* (RNO) and *Sus scrofa* (SSC)), the mean and median genomic size (in kb) of the groups and the maximum number of genes in the largest groups are indicated.

The gap between species gets even larger when considering functional annotations and gene expression information. The percentage of groups of genes used for gene expression comparisons fluctuates strongly between humans (94%) or mice (93%) and fish (24%) or horse (0%). Similar variations exist for functional annotations: 83% and 88% of duplicated genes in humans and mice are annotated by GO terms in the GOA database *versus* just 12% and 25% in chicken and pig groups (Table 1).

### Database Content Analyses

**Figure 3:**
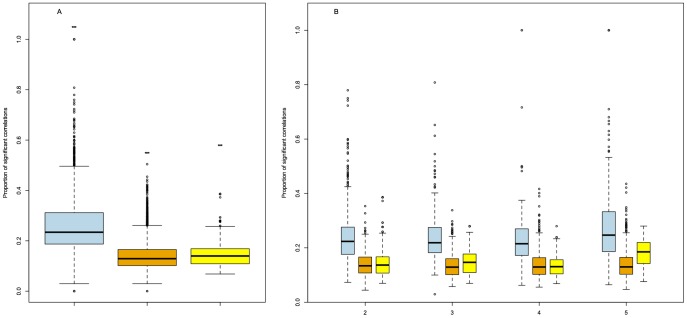
Proportion of significant correlations. Boxplots of significant correlations of expression for duplicated genes (blue), non-duplicated genes (orange) and randomly-selected genes (yellow). (**A**) Correlations for all groups of genes. Means with a different letter are significantly different according to Student’s R t-tests at *p*<0.05 (n = 3320, 2760 and 13605, respectively). (**B**) Correlations according to the number of genes within groups. For every group size, the means of each type of group are significantly different (p<0.05).

**Figure 4:**
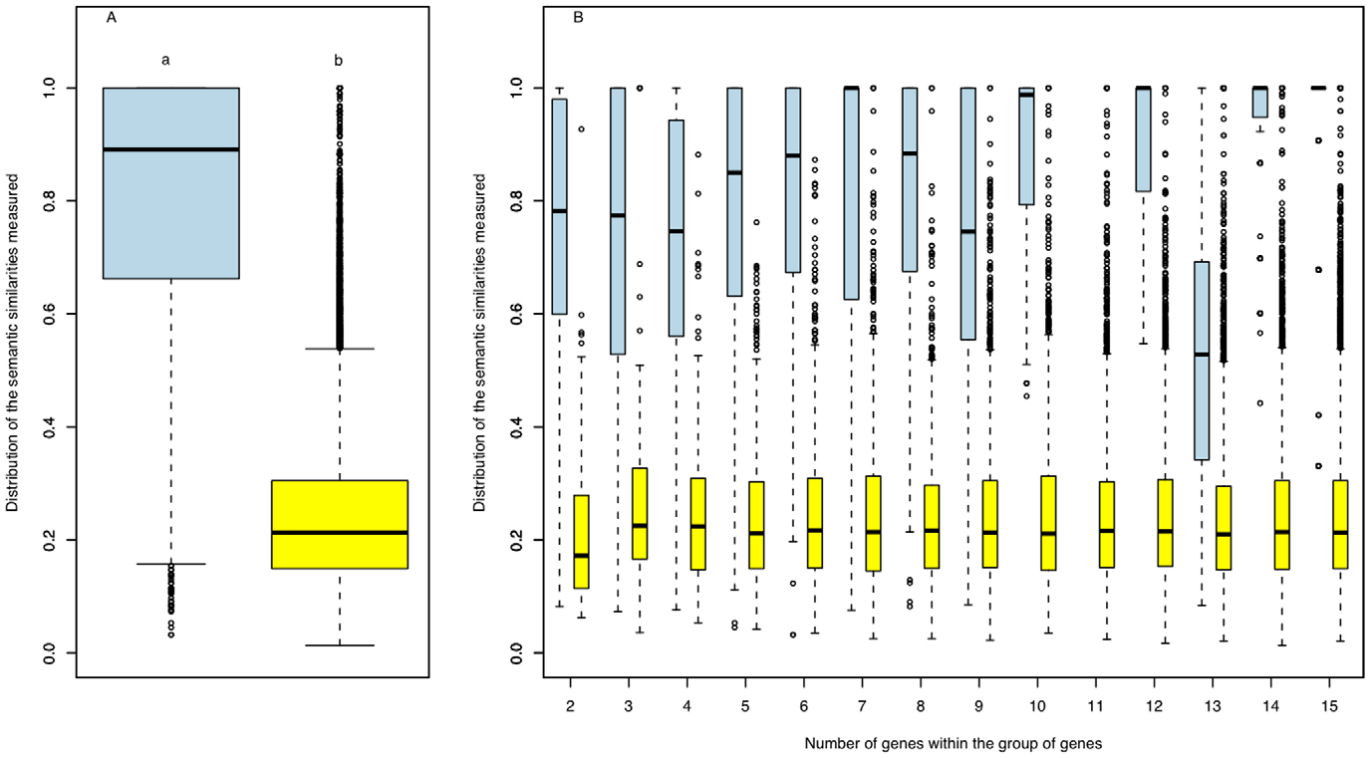
Distribution of semantic similarities. (**A**) Distribution of GO biological process semantic similarities in duplicated gene groups (blue) *vs.* randomly-selected gene groups (yellow). Means with a different letter are significantly different according to Student’s R t-tests at *p*<0.05. (**B**) Details of the same distribution with groups pooled by size. The mean of each duplicated group is significantly different from the mean of each randomly-selected genes group (*p*<0.05). *Note: no data were available for the group with 11 genes.*

The pairwise Pearson correlations on the gene expression and semantic similarity values of the groups of duplicated genes were characterised in humans (Figures 3 and 4) and compared to results obtained from non-duplicated co-localised genes or randomly selected genes. These gene expression analyses were led on groups of 5 or less genes, as expression data for larger groups is often too incomplete to enable meaningful analysis. The same approach was applied for the analysis of semantic similarities in GO annotations (GOA), but with a maximum of 15 genes per group. Interestingly, the proportion of significant correlation was higher in groups of duplicated genes than in co-localised non-duplicated genes or genes randomly selected on the genome (figure 3A). The same results were observed when analyses were performed according to size of the group (figure 3B). Note that the proportion of significant correlation is similar between co-localised non-duplicated genes and genes randomly selected on the genome. Similar results were observed on semantic similarities, with higher values for duplicated genes than for randomly-selected genes whatever the number of genes in the group (figure 4A and 4B). This was not only the result of a higher proportion of electronic annotations (IEA) inferred from sequence similarities between these duplicated genes. Indeed, although IEA proportion increased with the number of duplicated genes in the groups, it was far lower in humans, for which 76% of the groups have been annotated, and in mouse, which is another ‘well-annotated’ species (88%), than in relatively ‘poorly-annotated’ species’ such as ECA (42%) and SSC (at just 25%; see Table S1) in which most of the annotations are IEA (figure S1).

### Database Interface

DGD has a web GUI handling queries in two major sections ― the browse page and the search page. The browse page gives direct access to database content for a species, a specific chromosome, or a defined genomic region. The search page allows users to run database queries for different terms using specific gene ID (Ensembl, Uniprot, RefSeq, GenBank, among others…), chromosomal location (chr:start.end) or any keywords (e.g. GTPase, death, fatty acids, etc.) that are searched for in the gene description. Users can perform multiple queries by typing several of these terms into the input box or by uploading a text file with the terms to search. In all cases, the search can be performed across all species or limited to a specific species. The DGD website search engine runs the query in the whole Ensembl dataset and cross-references database, and displays all the results even if the genes are not included in any co-localised and duplicated groups.

When a specific group of duplicated genes is selected, each gene is described by name (HGNC), by chromosome and by base pair location. The proportion of experiments with significant correlation of expression and the semantic similarities between genes in biological process, molecular function and cellular component gene ontology terms are also shown as a graph if the information is available.

Cross-references can be added to this display (functional annotation, various gene IDs from others databases). Users should note that the lists of cross-references are species-dependent, and so this feature is disabled for queries across all the species. The display gives hyperlinks to the selected cross-reference databases.

For both browse pages and search pages, users can choose between different export formats or display modes (lists of genes or lists of groups, in tab-delimited file format).

DGD is publicly available as a SOAP web service that has been implemented in Java using the Opal2 toolkit [38]. The DGD web service only accepts Ensembl gene IDs as search input and cannot return external references directly. However, a second web service named Xref dedicated to cross-references management is available on the Genouest server [39]. For a given set of genes, the Xref web service searches corresponding Ensembl genes using cross-references, and returns a set of external references for the given set of genes. Thus, users should use the Xref web service in contexts when they need conversions between Ensembl gene IDs and other identifiers. Full developer documentation, WSDL files, code examples, and Taverna workflows are all available for both services via the DGD website.

## Discussion

The goal of the DGD database was to provide information on co-localised duplicated genes. To this end, two parameters had to be defined: the sequence similarity threshold between two genes, and the maximum distance defining duplicated genes as co-localised. The literature features various different approaches developed for detecting duplicated genes. Most of these approaches revolve around sequence comparisons using either FASTA [9], [16], [40] or BLAST [28], [41], [42]. The threshold values defined by these comparison tools are generally based on 1) a first selection based on an e-value threshold to remove non-relevant sequence comparison results, and 2) the value defined by Rost [43], who proposed a formula using percentage identity and length of the alignment between the two sequences. Note that some studies have only used the e-value and a minimum alignment coverage threshold [25], [42]. Here, we applied another approach first proposed by Li *et al.* [44] that computes another identity value *I’*, weighting the initial identity value with the number of amino acids and the length of the aligned region. This improvement avoids the clustering of non-homologous genes that share the same domain, such as when a short protein shares domains with a longer protein. The threshold values proposed by Li *et al*. were used to define the groups of pairwise duplicated genes (i.e. I’≥30% for alignment >150 aa and I’≥p’ from Rost for alignment <150 aa). Using these more stringent thresholds instead of those of the Ensembl database (2%–24%) results in a conservative approach that is expected to reduce the number of false-positives.

Another major parameter that dictates the definition of groups of duplicated genes is size of the gene window. In the literature, the maximum distance within which duplicated genes are considered as co-localised is defined using either a physical distance [22], [27] or a window including *n* genes [29], [30]. The physical distance approach may be more stringent but it has a major pitfall: as genome length and gene density are not the same in the different species, the distance has to be defined in a species-specific way (from 200 kb for *C. elegans* to 1 Mb for *H. sapiens*, for instance). The gene window approach, however, is compatible with many species and is not sensitive to gene density variability between chromosomes and between species. Here, duplications were searched within a window of 100 genes. Although at first sight this may seem a large number, the median size of the duplicated groups reported here was 105 kb in humans and was fairly similar in other species, with values ranging from 58 kb in chicken to 248 kb in horse. This suggests that the duplicated genes identified are closely localised, and that defining distance as a number of genes rather than a physical distance does not greatly affect the genomic size of the groups.

The total number of groups of duplicated genes differs between species (Figure 2). These differences are observed mainly in groups containing two or three duplicated genes and between mammalian species and other species. In mammals, the only exception is the pig, for which the genome assembly is of poor quality, which could lead to the identification of false-positive groups of duplicated genes. This artificially increases the number of small groups of duplicated genes. In chicken and zebrafish, part of the differences could be assigned to the phylogeny distance with mammals [45].

Every species featured some very large groups, ranging from 62 genes in GGA to 428 genes in DER. In humans, the largest groups include T-cell receptor genes, zing finger genes, immunoglobulin genes, or notoriously highly duplicated olfactory receptor genes [46]. In fact, it is possible to find clear false-positive groups due to errors in the genome assemblies, especially for most current genomes that, like the pig, are what Yandel and Ence (2012) called ‘standard draft assembly’ genomes [47]. However, as the DGD database is updated at each Ensembl update cycle, we expect to see genome assembly errors fixed in the future.

Gene co-expression level and functional similarity in GO annotations can be combined inside a group by computational processes on data from GEO and GOA. We thus tested a few hypotheses using the human data. The first and highly controversial hypothesis is that gene co-expression might be higher in groups of duplicated genes than in groups of randomly-selected genes [10], [11], [12], [13]. As illustrated in Figure 3A, co-localised duplicated genes have a higher proportion of significant co-expression than co-localised non-duplicated genes or genes randomly selected in the genome. This difference is observed whatever the number of genes within the groups (Figure 3B).

Another interesting hypothesis to test was whether there is functional conservation or divergence between duplicated genes [9]. Comparing GO semantic similarities between co-localised duplicated genes against randomly-selected genes revealed that annotated biological processes present much higher similarities between co-localised duplicated genes (Figure 4A). Surprisingly, the similarity between genes significantly increases with group size (Figure 4B). This is probably due to a lack of “specific” annotation when the number of duplicated genes does not allow experimental validations. Indeed, for most of the genes annotated in the large duplicated groups, the annotation was automatically inferred from electronic annotation (IEA evidence code). As shown in figure S1, this is particularly true in species for which annotation is qualified as “poor quality”, the best examples being ECA and SSC with 42% and 25%, respectively, of the groups annotated with almost all GO terms inferred electronically (IEA), but less so in model species (HSA, MMU, and to a lesser extent RNO) for which annotation is qualified as “good quality”. Taken together, these results clearly suggest that, at least in humans, tandem and multi-duplicated genes show higher co-expression levels and similarity of functional GO annotations than other genes.

### Conclusion

This database provides a simple way to quickly and easily find groups of tandem duplicates or large groups of multigene families by gene identifier, chromosomal location and/or keywords. Gene co-expression level and semantic similarities in functional annotations are also displayed when raw data is available. DGD is the first database to integrate this genomic information on co-localised duplicated genes with gene expression data and GO annotation similarity. This database can be readily expanded to other genomes as long as genomic annotations and peptide sequences are available.

## Materials and Methods

### Sequence Data

As shown in Figure 1, peptide sequences and chromosomal location of the genes were downloaded from the Ensembl FTP site [48] (Ensembl version 68) for 9 species: *Bos taurus* (BTA), *Danio rerio* (DER), *Canis familiaris* (CAF), *Gallus gallus* (GGA), *Equus caballus* (ECA), *Homo sapiens* (HSA), *Mus musculus* (MMU), *Rattus norvegicus* (RNO) and *Sus scrofa* (SSC). For each gene, only the longest peptide sequence was kept (peptide sequence numbers are given in Table 1).

### Identification of Duplicated Genes

Duplicated genes were identified using a two-step strategy. For each genome, a BLAST search was conducted between all peptide sequences of the genes in a chromosome. To determine whether two peptides were similar, we computed identity *I’ = I x Min(n_1_/L_1_,n_2_/L_2_)* proposed by Li *et al.* [44], where I is the proportion of identical amino acids in the aligned region (including gaps) between sequences 1 and 2, *L_i_* is the length of sequence *i*, and *n_i_* is the number of amino acids in the aligned region in sequence *i*. Two genes were considered duplicates if an all-against-all BLAST search within a window of 100 genes [29], [30] met the following criteria: i) e-value is ≤0.2 (only to filter non-relevant BLAST results); ii) I’ ≥30% if L ≥150 a.a. (where L is the length of the aligned region) or I ≥0.01n+4.8L^−0.32(1+exp(−L/1000))^ [43] if L <150 a.a. (where n = 6 as it makes the formula continuous at L = 150), as proposed by Li *et al.* [44]. Within the best BLAST hits for a given gene query, we selected the “hit” gene that had the closest chromosomal location downstream of the gene queried.

Duplicated gene groups were then put together based on the principle of a simple transitive link between the remaining genes: if gene A was similar to gene B and to gene C, then genes A, B and C were included in the same group, even if genes B and C were not found similar. Chromosomal location information and gene annotations (name and description) of each gene for all duplicated groups were then incorporated into a MySQL database.

### Database Objects

For each species, Ensembl cross-references [48] were integrated into the MySQL database to enable queries on specific genes using an Ensembl or HGNC keyword. In addition, data on Ensembl objects (genes, transcripts and translations) as well as other database objects (NCBI, etc.) were also collected to be displayable in the results page if needed. The list of available reference sources was specific to each species depending on the sources found in the Ensembl dataset. For each gene, the external references displayed are those associated to the gene and to any of its transcripts and any of the corresponding translations.

Functional gene annotations were retrieved from the Gene Ontology Annotation (GOA) database [49]. The GO structure used to compute similarity was obtained from the term and term2term tables of the GO database [50].

All database updating procedures have been incorporated into the BioMaj workflow engine [51] to integrate future updates at each new Ensembl database version.

### Gene Expression Correlations Using GEO

The HGNC id of each duplicated gene was searched through the annotation platform (GPL) of the Gene Expression Omnibus (GEO) database [52]. The corresponding GEO experiments (GSE) were extracted. Only GSE expression data that satisfied the following conditions were kept: a) a minimal number of 3 samples available; b) the genes of a duplicated group were all present within the GSE; c) GSE with null values or always the same value were discarded.

For each group of duplicated genes and for each GSE, the Pearson correlation and associated *p*-value were computed between each gene pair using a bilateral test, and the proportion of significant correlations for each gene pair within a group of duplicated genes was retrieved.

To assess whether co-localised duplicated genes had a higher proportion of significant correlations, we ran this same procedure on non-duplicated genes that were selected as i) co-localised or ii) randomly distributed among the human genome. The proportions of significant correlations between conditions were tested using a *Student* t-test.

### Similarities in GO Annotations

Semantic similarities in GO annotations were determined using Wang’s method [36] and computed pairwise in a group every time at least two annotated genes were found. As GO is split into three different branches – Biological Process, Molecular Function and Cellular Component – three similarity values were computed for each pairwise comparison. All the similarity values calculated with this method were bounded from 0 to 1. The higher the similarity value, the more the compared genes shared the same biological functions. Wang considers two genes as fairly similar at a similarity value of 0.5.

## Supporting Information

Figure S1 **Proportion of IEA according to duplicated gene number in the groups in nine species.**
(TIF)

Table S1 **Description of DGD groups annotated for Gene Ontology.** For each species, the number of groups, the number of annotated groups with GO terms and the percentage of groups annotated are indicated.
(DOC)
