# Supplementary figures and images for: The Duplicated Genes Database: Identification and Functional Annotation of Co-Localised Duplicated Genes across Genomes
Source: PLoS One. 2012 Nov 28;7(11):e50653. doi: 10.1371/journal.pone.0050653 (PMC3508997; doi:10.1371/journal.pone.0050653)

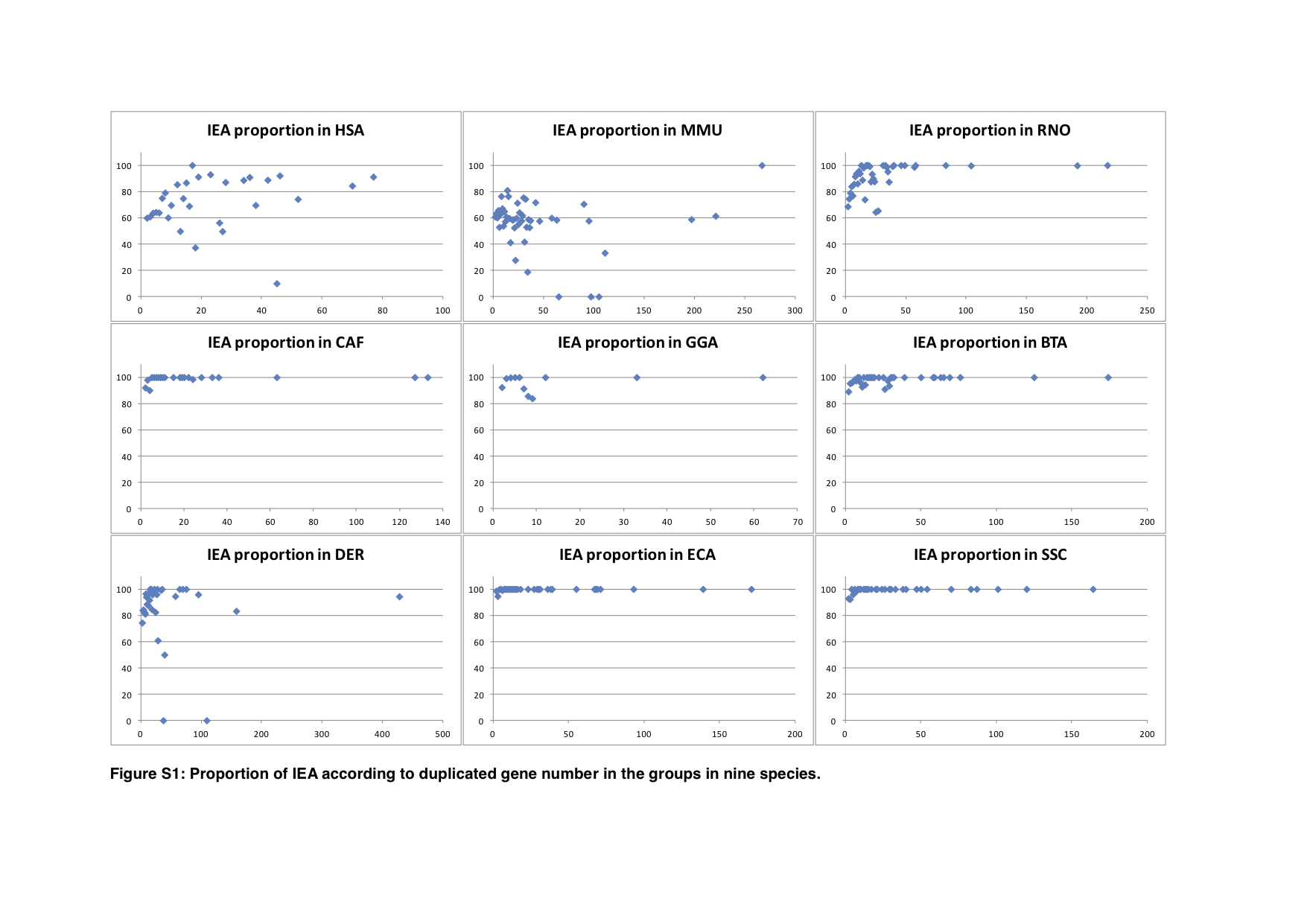

Supplement: Figure S1 — Proportion of IEA according to duplicated gene number in the groups in nine species. (TIF) [file pone.0050653.s001.tif]
