# Supplementary material for: The Duplicated Genes Database: Identification and Functional Annotation of Co-Localised Duplicated Genes across Genomes
Source: PLoS One. 2012 Nov 28;7(11):e50653. doi: 10.1371/journal.pone.0050653 (PMC3508997; doi:10.1371/journal.pone.0050653)
Supplement: Table S1 — Description of DGD groups annotated for Gene Ontology. For each species, the number of groups, the number of annotated groups with GO terms and the percentage of groups annotated are indicated. (DOC) [file pone.0050653.s002.doc]

Table S1

|  | HSA | MMU | RNO | CAF | GGA | BTA | DER | ECA | SSC |
| --- | --- | --- | --- | --- | --- | --- | --- | --- | --- |
| Group number | 964 | 994 | 959 | 736 | 444 | 798 | 1412 | 894 | 1210 |
| Annotated groups | 733 | 875 | 580 | 315 | 235 | 462 | 572 | 378 | 305 |
| Proportion % | 76 | 88 | 60 | 43 | 53 | 58 | 41 | 42 | 25 |
